# Urinary steroid profile in relation to the menstrual cycle

**DOI:** 10.1002/dta.2960

**Published:** 2020-11-20

**Authors:** Jenny Schulze, Tina Suominen, Helena Bergström, Magnus Ericsson, Linda Björkhem Bergman, Lena Ekström

**Affiliations:** ^1^ Karolinska Institute, Department of Laboratory Medicine, Division of Clinical Pharmacology C1:68 Karolinska University Hospital Stockholm Sweden; ^2^ Helsinki Doping Control Laboratory, Forensic Toxicology Unit Finnish Institute for Health and Welfare (THL) Helsinki Finland; ^3^ Department of Neurobiology, Care Sciences and Society (NVS), Division of Clinical Geriatrics Karolinska Institute Stockholm Sweden; ^4^ French Doping Control Laboratory, Agence Française de lutte contre le dopage (AFLD) Département des Analyses France; ^5^ Department of Clinical Pharmacology C1:68 Karolinska University Laboratory, Karolinska Hospital Stockholm Sweden

**Keywords:** ABP, doping in sports, menstrual cycle, steroid profile, T/E

## Abstract

The interpretation of the steroidal module of the Athlete Biological Passport (ABP) in female athletes is complex due to the large variation of the endogenous urinary steroids. The menstrual cycle seems to be one of the largest confounders of the steroid profile. The duration of the different phases in the menstrual cycle differs between women and is difficult to predict only by counting days after menstruation. Here, we have determined the follicle, ovulation, and luteal phases, by assessing the menstrual hormones in serum samples collected from 17 healthy women with regular menses. Urine samples were collected three times per week during two consecutive cycles to measure the urinary steroid concentrations used in the ABP.

The metabolite that was mostly affected by the menstrual phases was epitestosterone (E), where the median concentration was 133% higher in the ovulation phase compared to the follicle phase (*p* < 0.0001). The women with a large coefficient of variation (CV) in their first cycle also had a large CV in their second cycle and vice versa. The inter‐individual difference was extensive with a range of 11%–230% difference between the lowest and the highest T/E ratio during a cycle.

In conclusion, E and ratios with E as denominator are problematic biomarkers for doping in female athletes. The timing of the sample collection in the menstrual cycle will have a large influence on the steroid profile. The results of this study highlight the need to find additional biomarkers for T doping in females.

## Graphical abstract

The menstrual cycle is one of the largest confounders of the steroid profile in female athletes. We have determined the follicle, ovulation, and luteal phases, by assessing the menstrual hormones in serum samples in 17 women as well as determined the urinary ABP steroids for two consecutive cycles. The impact of the different phases of the menstrual cycle on the steroid profile and the correlation between menstrual hormones and urinary steroid are investigated.

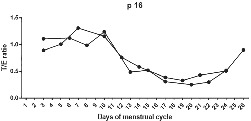

**Abbreviations**
5α‐Adiol: 5α‐androstane‐3α,17β‐diol
5β‐Adiol: 5β‐androstane‐3α,17β‐diol
A: androsterone
ABP: athlete biological passport
EAAS: endogenous anabolic androgenic steroid
E: epitestosterone
E2: estradiol
Etio: etiocholanolone
GC‐MS/MS: gas chromatography tandem mass‐spectrometry
LC‐MS/MS: liquid chromatography tandem mass‐spectrometry
LH: luteinizing hormone
P: progesterone
T: testosterone
WADA: World Anti‐Doping Agency

## 1 INTRODUCTION

In order to see if an athlete has used testosterone, or any other endogenous anabolic androgenic steroid (EAAS), the urinary steroid profile, including the concentrations of testosterone (T), and the four T metabolites androsterone (A), etiocholanolone (Etio), 5α‐androstane‐3α, 17β‐diol (5αAdiol), and 5β‐androstane‐3α,17β‐diol (5βAdiol), as well as epitestosterone (E), are analyzed by GC‐MS/MS. Five ratios (T/E, A/Etio, 5αAdiol/E, 5αAdiol/5βAdiol, and A/T) are longitudinally monitored in a urinary module of the athlete biological passport (ABP).^1^

The intra‐subject variations of all the urinary steroid metabolites and the ratios are larger in females than in males^2^ making the interpretation of female samples more challenging. It has been shown that T/E fluctuates during the menstrual cycle,^3^, ^4^ which was further investigated in six^5^ and nine^6^ women, respectively. E was higher in the luteal phase, and hence, the T/E ratio decreased throughout the menstrual cycle.^5^ However, in both these studies, the menstrual phases were interpreted based on the number of days after the first menstrual day. As this “day method” often do not corroborate with the actual menstrual phase, it will be more accurate to identify the biological phases of the menstrual cycle by hormonal analyses.^7^

A cycle starts with menstruation for 3–7 days, followed by a pituitary release of follicle stimulating hormone (FSH) and luteinizing hormones (LH) to promote development of follicles. The formed Graafian follicle secretes estradiol (E2), which peak just before mid‐cycle and stimulate a rise of LH leading to rupture of the Graafian follicle and the development into corpus luteum which secretes progesterone (P) in the later part of the cycle.^8^ The reason why urinary E increases in the later phase of the cycle is not known but may be associated with the increase of LH and P in the ovulation and luteal phase, respectively. We have seen that hormonal contraceptives (HC) mediated E changes exhibit strong correlation with the gonadotropins to a much larger extent than the other ABP metabolites,^9^ whereas no studies have investigated the correlation between urinary E and the other female hormones P and E2. Here, we aim to study if the concentrations of the urinary steroid metabolites differ between the different biological phases of the menstrual cycle. Moreover, the present study will provide an understanding if the intra‐individual fluctuation of the urinary steroid ABP‐ratios is similar between two menstrual cycles.

## 2 MATERIAL AND METHODS

### 2.1 Study population

**FIGURE 1:**
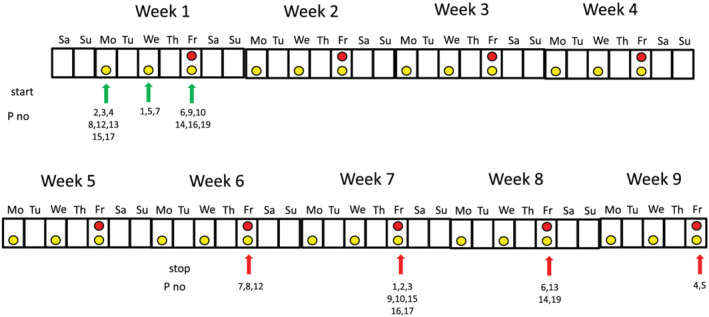
Illustration of the urine (yellow circles) and blood (red circles) samples collected from the 17 participants during two menstrual cycles. The green and red arrows show when the different participants started and ended the sample collections respectively [Colour figure can be viewed at wileyonlinelibrary.com]

Seventeen healthy women with regular menses not using hormones were included. Urine and blood samples were collected 3 times/week and 1 time/week, respectively, for two consecutive cycles; see Figure 1. The mean age and body mass index (BMI) for the subjects at inclusion were 33.2 ± 6.5 years and 21.6 ± 1.7 kg/m^2^. During the study, the mean number of bleeding days was 7, and cycle length was 24.5 days. For more details, see Mullen et al.^10^

Serum levels of E2, P, and gonadotropins were determined by radioimmunoassay using a commercial kit from Diagnostica, Basel, Switzerland. FSH and LH were determined by electrochemiluminescence immunoassay (ECLIA) using commercial kits from Roche Diagnostics AG, Malmö, Sweden. The follicle phase was defined as E2 < 81 pg/ml, P < 1.6 ng/ml, and low FSH and LH. Ovulatory phase was defined as E2 ≥ 81 pg/ml, P < 1.6 ng/ml, and LH higher than FSH. Luteal phase was defined as P > 5.3 ng/ml.^11^

Of the 17 participants, all had samples from all three phases in one or both menstrual cycles, that is, follicular, luteal, and ovarian phase. Twelve of the participants had samples from two complete menstrual cycles including the three phases: follicular, luteal, and ovarian phases according to the measured hormone levels in the samples. Five participants contributed with only one complete menstrual cycle including the three phases. To summarize, blood samples from 29 complete menstrual cycles, including follicular, luteal, and ovarian phases, could be retrieved from 17 women.

Morning urine were collected 3 times/week (Mondays, Wednesdays, and Fridays); see Figure 1. Between 18 and 27 urine samples were collected from each participant.

### 2.2 Urinary steroid analyses

The conjugated steroids were hydrolyzed using β‐glucuronidase and extracted by liquid‐liquid extraction (LLE) with diethyl ether at alkaline pH. The steroids were derivatized with a mixture of *N*‐methyl‐*N*‐(trimethylsilyl)trifluoroacetamide (MSTFA), trimethyliodosilane, and an antioxidant to trimethylsilyl (TMS) ethers and enol ethers. The urinary steroid profile was analyzed using a fast, comprehensive screening method for doping agents in urine by gas chromatography‐triple quadrupole mass spectrometry.^12^, ^13^

The limit of detection (LOD) and limit of quantification (LOQ) for T were 0.4 ng/ml and 0.88 ng/ml; E 0.4 ng/ml and 0.56 ng/ml; 5αAdiol 0.9 ng/ml and 3.4 ng/ml; 5βAdiol 1.1 ng/ml and 4.1 ng/ml; A 4 ng/ml and 57 ng/ml and Etio 4 ng/ml and 60 ng/ml, respectively.

### 2.3 Instrumentation

The steroids were chromatographically separated on a HP1 column (16 m × 0.20 mm, 0.11 μm film), Agilent Technologies, Santa Clara, CA, USA). A 7890A GC with a 7000 Triple Quadrupole Detector (Agilent Technologies, Santa Clara, CA, USA) was used for separation and detection using electron impact (70 eV) and selective reaction monitoring (SRM). Nitrogen was used as collision gas and helium as carrier gas (1 ml/min, constant flow mode). The injection temperature was 290°C, with the MS source at 310°C. Chromatography was performed using a temperature program for optimal separation: 0.2 min 100°C, ramp 70°C/min until 180°C, ramp 3°C/min until 210°C, ramp 5°C/min until 228°C, and finally ramp 65°C/min until 310°C.

The specific gravity of the samples was analyzed by a digital refractometer (UG‐1 from Atago).

All urinary steroid concentrations are expressed as the unconjugated plus the glucuronide conjugated fraction. The effect of the urine dilution was adjusted for by normalizing the concentrations to a specific gravity of 1.020.

### 2.4 Analysis of confounding factors

According to WADA TD2018EAAS,^14^ all urine samples have to be analyzed for factors that might have an impact on the steroid profiles (“confounding factors”), such as intake of alcohol (measured in urine as ethyl glucuronide, EtG), microbial contamination, diuretics, the administration of antifungal pharmaceuticals, and aromatase inhibitors. The confounding factors were analyzed by dilute‐and‐shoot LC‐MS/MS using positive and negative electrospray. The concentration of EtG was estimated using a single calibration point of 5 μg/ml. All concentrations of 5 μg/ml and above were reported in accordance with WADA TD2018EAAS.^14^

### 2.5 Data analyses

When calculating the steroid concentrations in the follicular, luteal, and ovarian phases, only the urine sample collected on the same day as the blood sample was collected and was used (Figure 1). The blood samples were used to determine in which phase the menstrual cycle was at the time. For each of the 17 study subjects, urine samples were separated in the different cycles according to the concentrations of E2, P, FSH, and LH (see criteria above). In each participant, an average of the steroid concentrations in the three different phases of the two menstrual cycles was calculated.

Six of the 17 women had non‐detectable amounts of testosterone in their urine, that is, below the LOD of 0.4 ng/ml. The participants with the undetectable testosterone concentrations were excluded from the statistical calculations when the testosterone concentrations and ratios including T in different menstrual cycle phases were compared as well as for the CV calculations and correlation analyses, as the method was not sensitive enough to detect changes in this metabolite.

Statistical analyses were performed using Graph Prism v8.3.0 software (San Diego, CA). D'Agostino and Pearson normality test was conducted to test for Gaussian distribution. When the data were not normally distributed, non‐parametric test was used. Repeated measures ANOVA followed by Tukey's multiple comparisons test (if normal distributed) or Friedman's test followed by Dunn's Multiple Comparison test (if non‐parametric distributed) were used to compare the different menstrual phases. All the correlations analyses were performed using Spearman's rank order. A *p* value < 0.05 was considered statistically significant.

## 3 RESULTS

### 3.1 Urinary steroid metabolite concentrations in relation to menstrual phases

**FIGURE 2:**
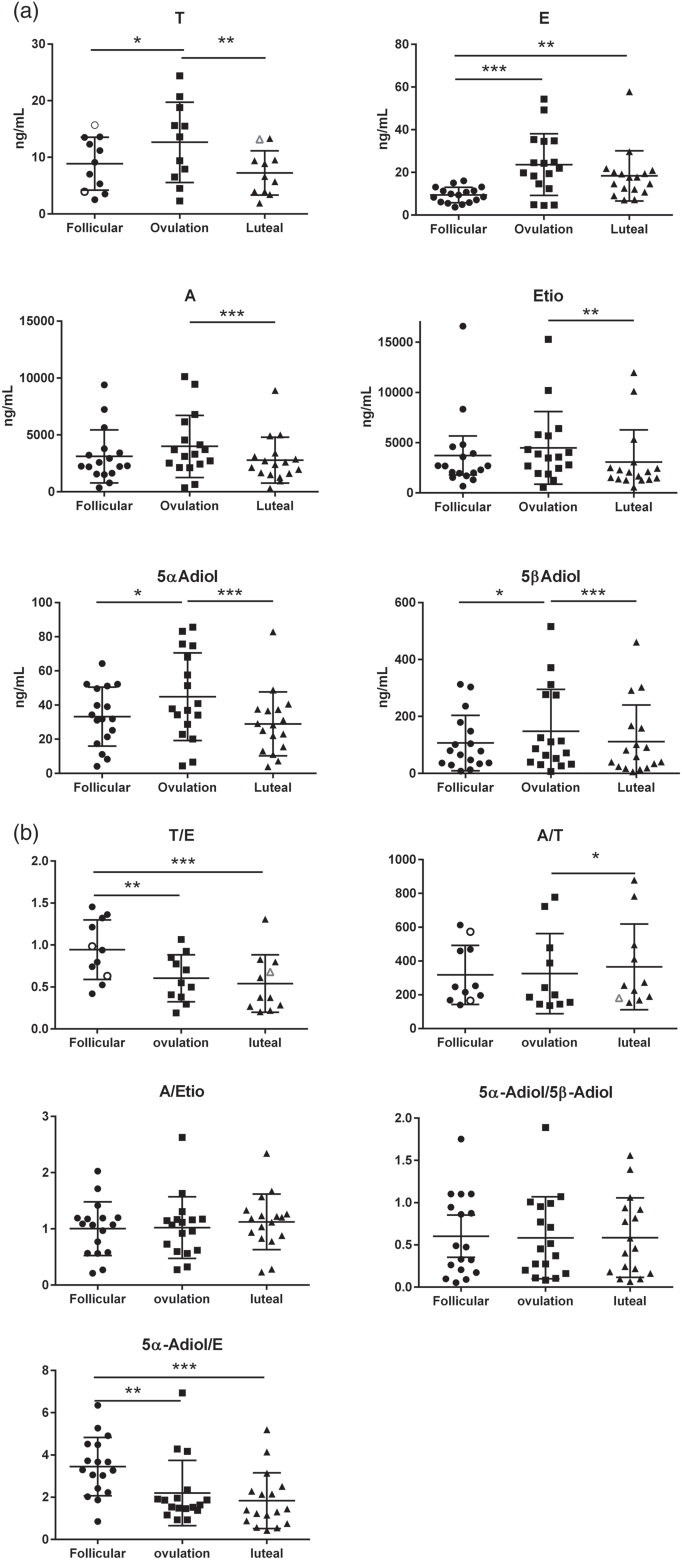
(a) The urinary concentrations of steroid metabolites in the different menstrual cycles in 17 individuals, except for T where *n* = 11. The dots represent the mean steroid concentration in the urine samples collected during the different menstrual phases, respectively, in each individual. The white dots represent participants where one of the measurements contain >5μg/ml EtG. The stars represent statistical significance between the menstrual phases (repeated measures ANOVA or Friedman's multiple comparison test) with * if *p* < 0.05; ** if *p* < 0.01, and *** if *p* < 0.001. (b) The urinary ratios of steroid metabolites in the different menstrual cycles in 17 individuals, except for T where *n* = 11. The dots represent the mean steroid ratio in the urine samples collected during the different menstrual phases respectively in each individual. The white dots represent participants where one of the measurements contain >5 μg/ml EtG. The stars represent statistical significance between the menstrual phases (repeated measures ANOVA or Friedman's multiple comparison test) with *p < 0.05; *p < 0.01, and ***p < 0.001

Samples from all three phases were collected and included in the statistical analyses. All concentrations of urinary ABP steroids were significantly changed during a menstrual cycle (Figure 2a). The steroid concentration that was most affected during a menstrual cycle was E, where the median value was 133% higher in the ovulation compared with the follicle phase (*p* < 0.0001). Consequently, the ratios with E in the denominator were mostly affected with T/E and 5αAdiol/E being significantly higher in the follicular phase with a median of 0.94 and 3.3, respectively, compared with the luteal phase where the median was 0.38 and 1.4 (*p* < 0.0001 for both) (Figure 2b). For the other metabolites, significantly higher concentrations were observed in the ovulation phase as compared with the luteal phase, but the other ratios were not affected to a high degree.

### 3.2 Confounding factors

**FIGURE 3:**
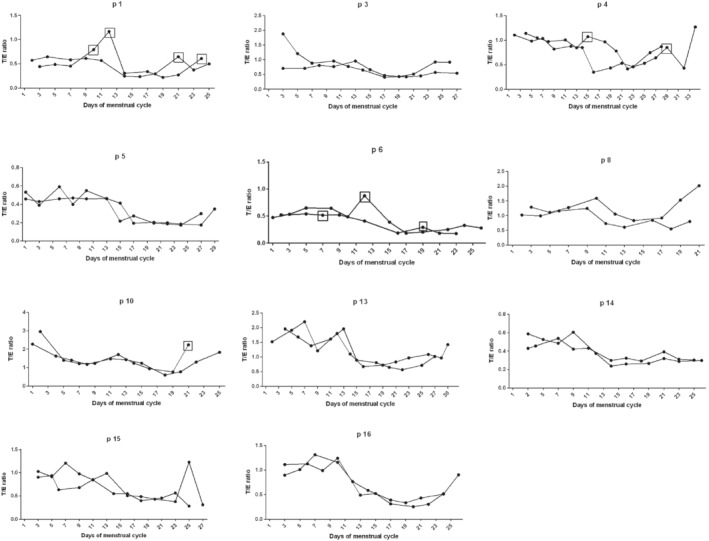
The T/E ratio during two cycles in the different study participants. The participants with no measurable levels of urinary T are not shown. The dots with a square illustrate samples with EtG levels >5 μg/ml

EtG (5.6–88.7 μg/ml) was found in 19 of the 368 urine samples. Of these, two were from a follicular phase and 1 was from a luteal phase included in the statistical analysis. Nine of the samples with EtG were from participants with undetectable T concentrations. As EtG may be associated with increased T concentrations,^15^, ^16^ the symbols showing the average T concentrations or T/E ratio from the participants with EtG concentrations in Figures 2 and 3 are white to illustrate that these values may be confounded by ethanol consumption. Removing these values from the average T concentration calculation did not change the significance level of differences in neither T concentration nor T/E ratio.

No other confounding factors were found in the urine samples.

### 3.3 Correlations between serum hormones and urinary steroid metabolites

In order to further study the urinary steroid profile in relation to the menstrual hormones, Spearman correlation analyses were conducted using all the data points from both the cycles (*n* = 114) (Table 1). It was evident that E was the urinary metabolite showing strongest correlation to the menstrual hormone E2, and the only metabolite correlated to P. The two 5β‐metabolites Etio and 5βAdiol did not correlate with any of the hormones, while A, as the only metabolite, correlated weakly with FSH (Table 1).

**TABLE 1:** Correlations between urinary ABP metabolites and serum levels of E2, P, LH, and FSH

	E2	P	LH	FSH
T (*n* = 11)	0.25*	−0.18	0.31**	0.19
E (*n* = 17)	0.60****	0.35****	0.31***	0.04
A (*n* = 17)	0.14	−0.08	0.18	0.22*
Etio (*n* = 17)	0.13	−0.12	0.12	0.17
5β‐Adiol (*n* = 17)	0.08	−0.17	0.15	0.10
5α‐Adiol (*n* = 17)	0.06	−0.18	0.26**	0.20*

*Note*: Values of Spearman's rank correlations are given.

* *p* < 0.05.

** *p* < 0.01.

*** *p* < 0.001.

**** *p* < 0.00001.

When the correlations were tested in each cycle phase, E was still strongly correlated with E2 in all phases: follicle phase Rs = 0.6, *p* < 0.0001, ovulatory phase Rs = 0.4, *p* < 0.01, luteal phase Rs = 0.6, *p* < 0.001. The E correlations with P and LH remained significant only in the ovulatory phase (Rs = 0.6, *p* < 0.0001 and Rs = 0.4, *p* < 0.05, respectively.

### 3.4 Intra‐subject variations of ABP ratios between two menstrual cycles

In Figure 3, the individual T/E ratio during two consecutives menstrual cycles is presented. The fluctuation pattern of the T/E ratio between the two cycles is very similar. The same relationship between two cycles was also found for the other ABP ratios (Figure S1). The median CVs for T and E and each ABP‐ratio are shown in Table 2. Ratios with E as denominator (T/E and 5α‐Adiol/E) have the highest CVs. The women with a high CV in their first cycle most often had a high CV in their second cycle and vice versa. There was no correlation between high CVs and low concentrations of T and E (not shown) indicating that the large CVs were an effect of individual variation rather than an analytical problem.

**TABLE 2:** Median of CV (%) of urinary ratios used in the steroid passport during two consecutive menstrual cycles in 17 participants

	Median CV cycle 1 (range)	Median CV cycle 2 (range)
T (*n* = 11)	39.6% (9.3–68.2)	34.4% (24.0–64.7)
E (*n* = 17)	54.1% (24.7–111)	48.6% (26.2–88.3)
T/E (*n* = 11)	42.8% (27.2–56.4)	36.5% (21.3–56.3)
A/T (*n* = 11)	18.1% (13.6–30.7)	18.4% (13.4–31.9)
A/Etio (*n* = 17)	15.5% (9.2–26.8)	15.6% (9.3–32.4)
5α‐Adiol/5β‐Adiol (*n* = 17)	20.3% (8.7–33.2)	19.1% (11.6–52.5)
5α‐Adiol/E (*n* = 17)	50.8% (30.9–65.7)	45.3% (29.7–67.0)

*Note*: Every participant has collected 9–14 urine samples per cycle depending on the length of the cycle (see Figure 1). Participants with T concentrations below LOD (0.4 ng/ml) were excluded in the calculations including T.

## 4 DISCUSSION

In this study, we have monitored the urinary steroid profile for two consecutive menstrual cycles. We found that the fluctuation pattern in urinary steroid concentrations and ratios repeats itself during the two cycles in the women included in the study. The reason why some women show large intra‐individual variations in the urinary steroid concentrations throughout their cycles while some women barely have any fluctuation at all may be related to the individual variability in ovarian steroid levels.^17^ Large CVs throughout the menstrual cycle will generate wider individual thresholds in the steroidal module of the ABP, which may be associated with greater complexity to detect doping with EAAS. The inter‐individual difference in the variation was extensive, with a range of 11%–230% difference between the lowest and highest T/E ratio during a cycle. For 5αAdiol/E, the range was 29%–460%. This large inter‐individual variation is in agreement with a large register study conducted in 3,229 female athletes.^2^

In agreement with our previous pilot study of six women,^5^ urinary E concentration was highest in the later part of the cycle, mimicking the hormonal fluctuation profile of E2 during the menstrual cycle. In fact, here, we show for the first time that urinary E strongly correlates with serum E2, through the whole menstrual cycle as well as in the different menstrual phases. E has been shown to exert anti‐androgenic properties in animal studies, and it is possible that E amplifies the estrogenic effects.^18^, ^19^ This theory is supported by the marked increase in urinary levels of E (but not the other ABP metabolites) observed during pregnancy.^20^

Interestingly, E was the urinary metabolite correlating with P serum levels to a much larger extent than the other urinary steroids. This corroborates with the hypothesis that E, like P, originates from pregnenolone.^21^ The biosynthesis of E is not fully elucidated, but it is believed to occur via the intermediate 5‐androstene‐3β,17α‐diol metabolized by CYP17A1.^21^ This is supported by an association between CYP17A1 single nucleotide polymorphism and E concentrations^22^ and the fact that co‐regulation of E and 5‐androstene‐3β,17α‐diol are down‐regulated in same fashion post T administration in males.^23^ However, the reason for the absence of urinary E and serum P correlation in the luteal phase when the highest amounts of P (and pregnenolone) is produced^24^ and is unknown.

Hormonal contraceptives have been shown to be an important confounding factor on the urinary steroid profile in female athletes.^9^, ^23^, ^25^ During the follicular phase, the urinary E concentration was suppressed in all the women in the study (Figure 2a). The suppression of E was almost as profound as seen in women taking hormonal contraceptives.^9^, ^23^, ^25^ Hormonal contraceptives are not one of the confounding factors that the laboratories have to analyze and report according to TD2018EAAS.^14^ The findings of this study support the decision not to include HC in the technical document, since it will be very difficult to distinguish between an atypical steroid passport finding due to hormonal contraceptive intake and the follicular phase of the menstrual cycle.

We noted that the urinary concentrations of T and its metabolites, that is, 5αAdiol, 5βAdiol, Etio, and A, differed between the menstrual phases, being highest in the ovulatory phase. In the ovulatory phase, some studies have reported an increase in serum T,^26^ which may explain the increase in the urinary rate of T and its metabolites. The dissimilarity in excretion pattern between E and T further indicates different synthetic and/or elimination pathways. All steroids are excreted mainly as glucuronides,^27^ except E which is also subject to sulphate conjugations.^28^, ^29^ The proportion of sulfate conjugated E compared with glucuronide conjugated E did not differ throughout the menstrual cycle^5^; that is, there seem to be no alteration in excretion routes during the menstrual cycle. It is possible that sulfate metabolites may be useful additional biomarkers of T doping, but notably, all studies available have been conducted in men only.^28^, ^30^, ^31^

Six out of the 17 women (35%) had undetectable T glucuronide levels in their urine samples. This may be due to a double deletion of the UGT2B17 gene coding for the major enzyme responsible for glucuronidation of T.^32^ As T was undetectable in all the >120 urine samples in these six women, it is likely that all of them carry the UGT2B17 del/del polymorphism. Unfortunately, we did not collect DNA for genotyping. Since the impact of the UGT2B17 deletion polymorphism is plausibly larger than the impact of the menstrual cycle on the urinary steroid levels, these six volunteers were excluded from calculations including testosterone concentrations.

In this study, we found EtG concentrations of >5 μg/ml in 5% of the samples. When excluding these samples from the calculations, the level of significance of the differences between the menstrual phases did not change. Hence, we believe that the EtG only had minor impact on the results in this study. Nine of the samples with reported EtG concentrations were from one individual (p 17) who had T concentrations < LOD and most likely did not have an effect of the results. All the samples that may have been confounded by ethanol are marked with white dots, or squares around the dots in the figures. Notably, previous studies have shown that EtG concentrations <5 μg/ml also can exert effect on the urinary steroid profile and that 5αAdiol is sometimes also affected.^15^

In addition to the large intra‐individual variation seen in E concentrations in women, another gender dilemma is that after T administration, urinary E excretion is not down‐regulated in women in contrary to men, regardless of UGT2B17 genotype.^33^, ^34^ Consequently, T administration in women is not necessarily associated with elevated T/E.^35^, ^36^ In addition to monitoring the urinary T/E ratio, future testing programs should consider monitoring serum T and/or other serum markers,^37^ which may improve the chance to identify women doping with T.^35^

In conclusion, we have investigated the extent of the variation of urinary steroids during the menstrual cycle. The steroid that is most affected by the hormonal fluctuations during the cycle is E, which is on average 133% higher during ovulation compared to the luteal phase. We show that the variation is fairly constant during two menstrual cycles, but the inter‐individual variation is large with some women showing hardly any variation, while others have extensive variation throughout the cycle. A more sensitive biomarker for T doping in females than E, for example, serum T is desirable.

## Supporting information


**Figure S1** A. 5αAdiol/E ratio. B. 5αAdiol/5βAdiol. C. A/Etio. D. A/T (women with T < LOD of 0.4 ng/mL excluded)
