# Supplementary figures and images for: Urinary steroid profile in relation to the menstrual cycle
Source: Drug Test Anal. 2020 Nov 20;13(3):550–7. doi: 10.1002/dta.2960 (PMC7984021; doi:10.1002/dta.2960)

1. **5αAdiol/E ratio**


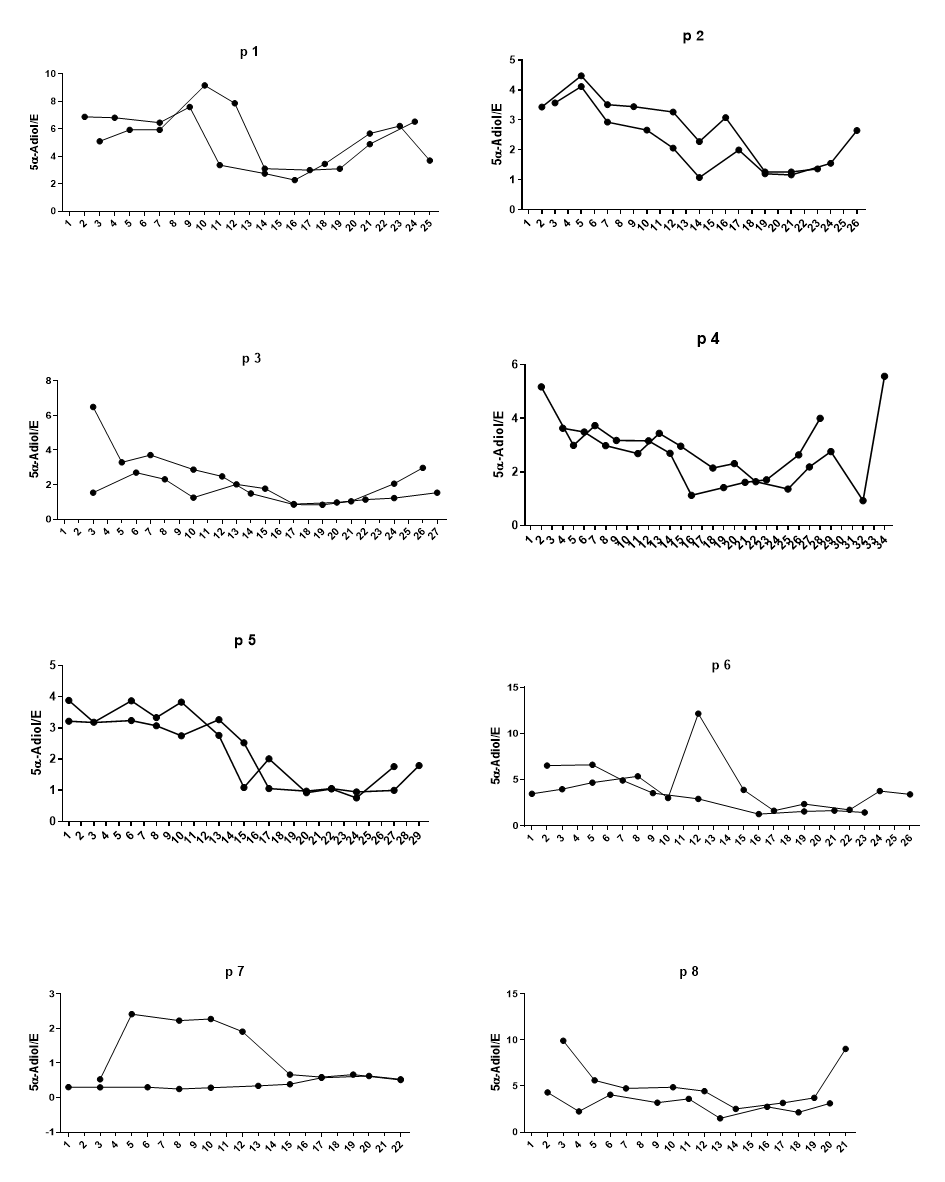


1. **5αAdiol/5βAdiol**

1. **A/Etio**

1. **A/T (women with T < LOD of 0.4 ng/mL excluded)**

Supplement: Supplementary file 1 — Figure S1 A. 5αAdiol/E ratio. B. 5αAdiol/5βAdiol. C. A/Etio. D. A/T (women with T < LOD of 0.4 ng/mL excluded) [file DTA-13-550-s001.docx]
